# BET Bromodomain Suppression Inhibits VEGF-induced Angiogenesis and Vascular Permeability by Blocking VEGFR2-mediated Activation of PAK1 and eNOS

**DOI:** 10.1038/srep23770

**Published:** 2016-04-05

**Authors:** Mingcheng Huang, Qian Qiu, Youjun Xiao, Shan Zeng, Mingying Zhan, Maohua Shi, Yaoyao Zou, Yujin Ye, Liuqin Liang, Xiuyan Yang, Hanshi Xu

**Affiliations:** 1 Department of Rheumatology, The First Affiliated Hospital, Sun Yat-sen University, Guangzhou, Guangdong, China; 2 Department of Anesthesia, Xiangya Medical School, Central South University, Changsha, China

## Abstract

The tyrosine kinase receptor vascular endothelial growth factor receptor 2 (VEGFR2) is a critical modulator of angiogenesis. Increasing evidence indicate the important role of bromodomain and extra-terminal domain (BET) of chromatin adaptors in regulating tumor growth and inflammatory response. However, whether BET proteins have a role in angiogenesis and endothelial permeability is unclear. In this study, we observed that treatment with JQ1, a specific BET inhibitor, suppressed *in vitro* tube formation of human umbilical vein endothelial cells (HUVECs) and *in vivo* angiogenesis in a Matrigel plug and oxygen-induced retinopathy neovascularization. JQ1 attenuated the VEGF-induced decrease in TEER in HUVECs and prevented Evans blue dye leakage in the VEGF-induced Miles assay in athymic Balb/c nude mice. BET inhibition with JQ1 or shRNA for Brd2 or Brd4 suppressed VEGF-induced migration, proliferation, and stress fiber formation of HUVECs. Furthermore, BET inhibition suppressed phosphorylation of VEGFR2 and PAK1, as well as eNOS activation in VEGF-stimulated HUVECs. Inhibition with VEGFR2 and PAK1 also reduced migration and proliferation, and attenuated the VEGF-induced decrease in TEER. Thus, our observations suggest the important role of BET bromodomain in regulating VEGF-induced angiogenesis. Strategies that target the BET bromodomain may provide a new therapeutic approach for angiogenesis-related diseases.

Angiogenesis plays a critical role in development and wound healing as well as in numerous pathological states such as cancers and rheumatoid arthritis^1^,^2^. Angiogenesis is a multi-step process that depends on endothelial cell (EC) proliferation and migration into the surrounding tissue and differentiation into new capillary tubes. Migration and proliferation of endothelial cells in response to angiogenic growth factors such as vascular endothelial growth factor (VEGF) play a crucial role in this process^3^. VEGF-mediated stimulation of vascular endothelial growth factor receptor 2 (VEGFR2), a type II transmembrane tyrosine kinase receptor expressed on endothelial cells and on circulating bone marrow-derived endothelial progenitor cells, results in activation of several signaling pathways, which regulate a variety of endothelial functions including endothelial survival, migration, permeability, and proliferation^4^,^5^,^6^. However, the precise mechanisms underlying this process remain unclear.

The cytoskeleton, which directly modulates an impressive array of cell functions including cell division, cell shape maintenance, cell motility and differentiation, and intracellular signal transduction, plays an important role in angiogenesis and permeability. The Rho GTPases, including RhoA, Rac1, and Cdc42, are critical regulators for alteration of the actin cytoskeleton^7^. The p21-activated kinases (PAKs), a family of serine/threonine kinases conserved from yeast to humans, are direct downstream effector molecules of Rho GTPases, Rac1, and Cdc42^8^. PAKs are subdivided into 2 groups, PAK1-3 (Group I) and PAK4-6 (Group II), based on structural organization and mode of regulation^9^, and undergo autophosphorylation on multiple sites^10^,^11^. PAKs play important roles in regulating cell motility, signal transduction, cell death, and survival^9^,^12^. It has been shown that normal PAK1 activity was required for endothelial migration and permeability^13^,^14^, and PAK1 inhibition by the autoinhibitory domain of PAK1 also suppressed angiogenesis^15^. In addition, endothelial nitric oxide synthase (eNOS), which generates nitric oxide (NO), also plays an important role in mediating angiogenesis and vascular permeability^16^.

Bromodomain and extra-terminal domain (BET) proteins (Brd2, Brd3, Brd4, and Brdt) are a family of epigenetic adaptors that bind to acetylated lysine residues, and thereby connect acetylated chromatin and gene transcription^17^. Pharmacological inhibitors of the BET protein family, including JQ1, exhibit anti-tumor activity in a range of malignancies^18^,^19^,^20^,^21^. BET proteins also recruit transcriptional coactivators, such as positive transcription elongation factor b (P-TEFb), to promote gene transcription in inflammatory conditions^22^,^23^,^24^,^25^. These studies indicate that targeted inhibition of the BET bromodomain may represent a novel therapeutic approach for some cancers and inflammatory disorders. However, little is known about the role of the BET bromodomain in vascular disorders. Although a recent report has shown that Brd4 is involved in endothelial inflammation and atherogenesis^26^, the contribution of the BET bromodomain to angiogenesis and permeability is still unclear. Therefore, in this work, we investigate the role of BET proteins in regulating angiogenesis and permeability, and the underlying mechanisms.

## Results

### BET bromodomain inhibition suppresses angiogenesis

**Figure 1:**
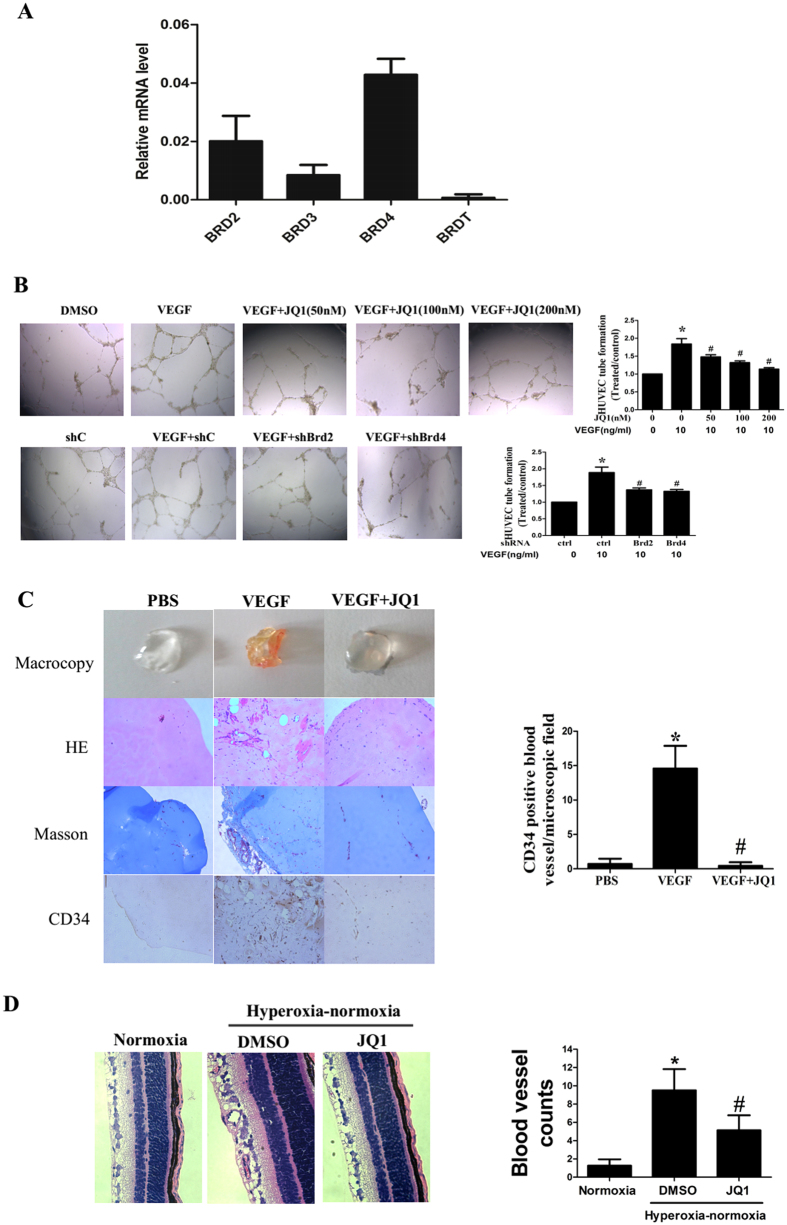
Expression of Brd in HUVECs and effect of BET bromodomain inhibition on angiogenesis. **(A**) Brd mRNA expression in HUVECs. Brd mRNA expression was measured by qRT-PCR analysis. The data represent the mean (S.E.M.) from 3 independent experiments. (**B**) Effect of BET inhibition on VEGF-induced *in vitro* tube formation of HUVECs. HUVECs, which were transfected with or without specific Brd2 shRNA (shBrd2) or Brd4 shRNA (shBrd4) or control shRNA (shC), were suspended in serum-free medium and seeded onto the Matrigel. They were pretreated with or without dimethyl sulfoxide (DMSO, as a control) or various concentrations of JQ1 for 6 h and then stimulated with or without VEGF (10 ng/mL) for 6 h. The left panel shows representative images of decreased tube formation in JQ1 treatment compared with control cells from at least 3 independent experiments. The right panel shows quantitative analysis of the number of intact tubes from 3 microscopic fields per well (n = 4). (**C**) Effect of JQ1 on *in vivo* angiogenesis using a Matrigel plug assay. Matrigel (500 μL/plug) was injected subcutaneously into 7–8-week-old male null mice. Mice were then intraperitoneally treated daily with vehicle (DMSO) or JQ1 (50 mg/kg·d) for 10 d. The Matrigel plugs were embedded with paraffin. Next, 5 μm sections were stained with H&E, Masson’s trichrome stain (endothelial cells stain red and Matrigel stains blue), or CD34 immunohistochemistry. Photographs of representative Matrigel plugs show vessel growth and CD34 expression (left panel). n = 6 for each experimental group. The right panel shows quantitative analysis of CD34. (**D**) Effect of JQ1 (50 mg/kg·d, i.p) on retinal neovascularization in the retinopathy-of-prematurity model. Serial cross sections of eyes were stained with H&E and blood vessels in the retina were quantified by light microscopy under 400× magnification (right panel). Scale bars: 100 μm. All values represent mean ± SD. *P < 0.05 vs. DMSO or shC or Normoxia, ^#^P < 0.05 vs. VEGF or VEGF + shC or DMSO.

The inhibitory effect of BET suppression on endothelial inflammation^26^ led us to investigate whether BET proteins are involved in angiogenesis. We first examined the expression patterns of BET proteins in endothelial cells. Analysis of HUVECs by qRT-PCR revealed that Brd2, Brd3, Brd4, and Brdt were transcribed, with Brd4 emerging as most highly expressed (Fig. 1A).

To evaluate the role of BET proteins in regulating angiogenesis, we first assessed the effect of BET inhibition on *in vitro* tube formation of HUVECs. As expected, JQ1 treatment inhibited VEGF-induced tube formation (Fig. 1B). To rule out a nonspecific effect of the small molecule inhibitor, we utilized the RNA interference technique to selectively reduce Brd expression. To rule out nonspecific interference, we constructed 3 different sequences of shRNA oligonucleotides for Brd2 or Brd4. As shown in Fig. S1, transfection with all 3 shRNA oligonucleotides downregulated endogenous Brd2 or Brd4 protein expression; however, the inhibitory effect of Brd2 shRNA-3 or Brd4 shRNA-3 was the most prominent. Accordingly, Brd2 shRNA-3 (Brd2 shRNA) or Brd4 shRNA-3 (Brd4 shRNA) was used for subsequent experiments. After 72 h of transfection, the cells were seeded onto the Matrigel. Transfection with Brd2 or Brd4 shRNA decreased VEGF165-induced tube formation compared with the control shRNA (Fig. 1B) and confirmed results obtained with JQ1.

Next, we evaluated the effect of JQ1 on *in vivo* angiogenesis using a mouse Matrigel plug assay. As shown in Fig. 1C, JQ1 inhibited VEGF-induced vessel growth and CD34 expression in a Matrigel plug. We also explored the effect of BET inhibition on hyperoxic-induced retinal neovascularization. Exposure of neonatal mice to hyperoxic conditions followed by normoxia causes retinal neovascularization^28^. As shown in Fig. 1D, hyperoxia induced more neovessels in the retina; however, JQ1 administration markedly suppressed retinal neovascularization, which indicates that JQ1 strongly inhibited angiogenesis *in vivo*.

### BET bromodomain inhibition suppresses vascular hyperpermeability

**Figure 2:**
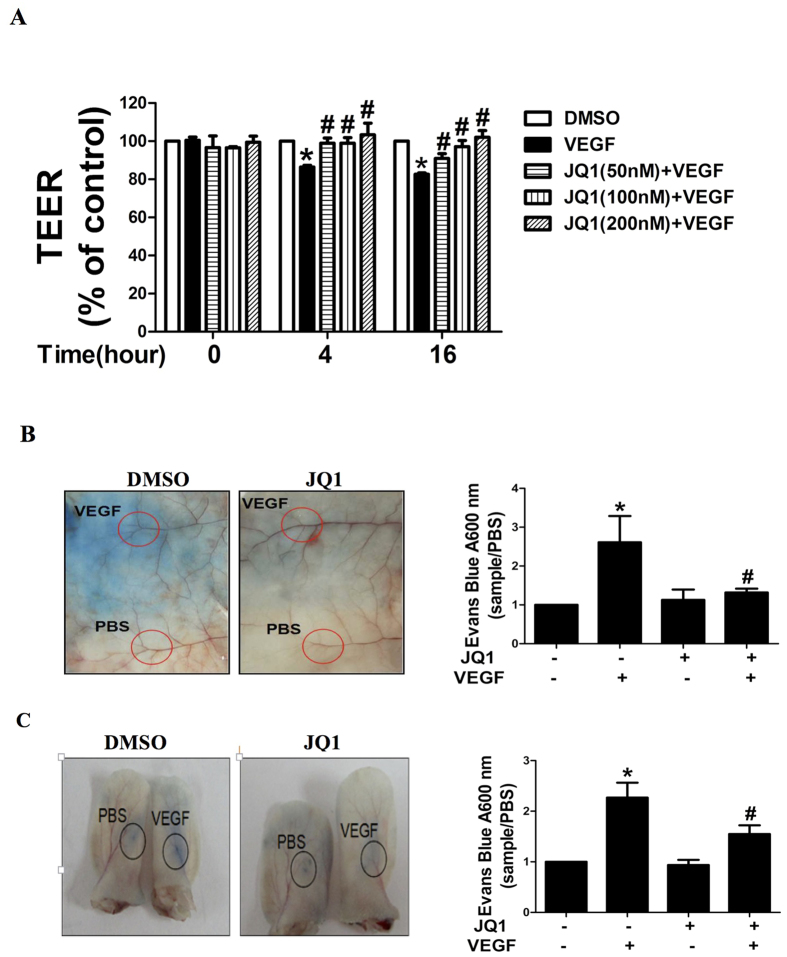
Effect of the BET bromodomain inhibitor JQ1 on vascular hyperpermeability. **(A**) Electrical resistance of a monolayer of HUVECs was measured by TEER. The cells were cultured to confluence in 24-well Transwell clear polyester membrane cell culture chambers, treated with various concentration of JQ1 or 0.1% DMSO for 6 h, and then stimulated with VEGF (10 ng/mL) or PBS for 4 h or 16 h. The experiments were carried out in triplicate in 3 separate experiments. (**B,C**) The BET inhibitor JQ1 suppressed VEGF-induced vascular permeability in the Miles assay in athymic Balb/c nude mice. Mice were pretreated intradermally with DMSO (as a control) or JQ1 (50 mg/kg·d) for 5 d. VEGF (100 ng in 50 μL/site) or PBS was injected intradermally in the dorsal skin and the ear. Mice were killed and skin samples (**B**) and ears (**C**) were dissected, photographed, and placed in formamide at 56 °C for 48 h to extract Evans blue. The amount of Evans blue in each sample was read by spectrophotometry. All values represent mean ± S.E.M. *P < 0.05 vs. control, ^#^P < 0.05 vs. VEGF.

Increased vascular permeability is critical for leukocyte transendothelial migration towards inflamed areas and angiogenesis. To assess the functional involvement of BET proteins in VEGF-induced EC barrier dysfunction, we first determined changes in TEER, a highly sensitive *in vitro* assay of permeability, in HUVECs pretreated with 0.1% DMSO (as control) or various concentration of JQ1. As shown in Fig. 2A, JQ1 treatment markedly attenuated the VEGF-induced decrease in TEER in HUVECs. Next, we investigated the effect of BET inhibition on vascular permeability *in vivo* using the Miles assay in athymic Balb/c nude mice. In this model, intradermal injection of VEGF causes leakage of Evans blue dye from the circulation into tissue. Intradermal administration of VEGF induced dye leakage within 60 min. Dye leakage was prevented by prior intradermal administration of JQ1 (Fig. 2B,C). These data clearly indicate an important role for BET proteins in mediating endothelial permeability.

### BET bromodomain inhibition suppresses migration, cytoskeleton alterations, and proliferation of HUVECs

**Figure 3:**
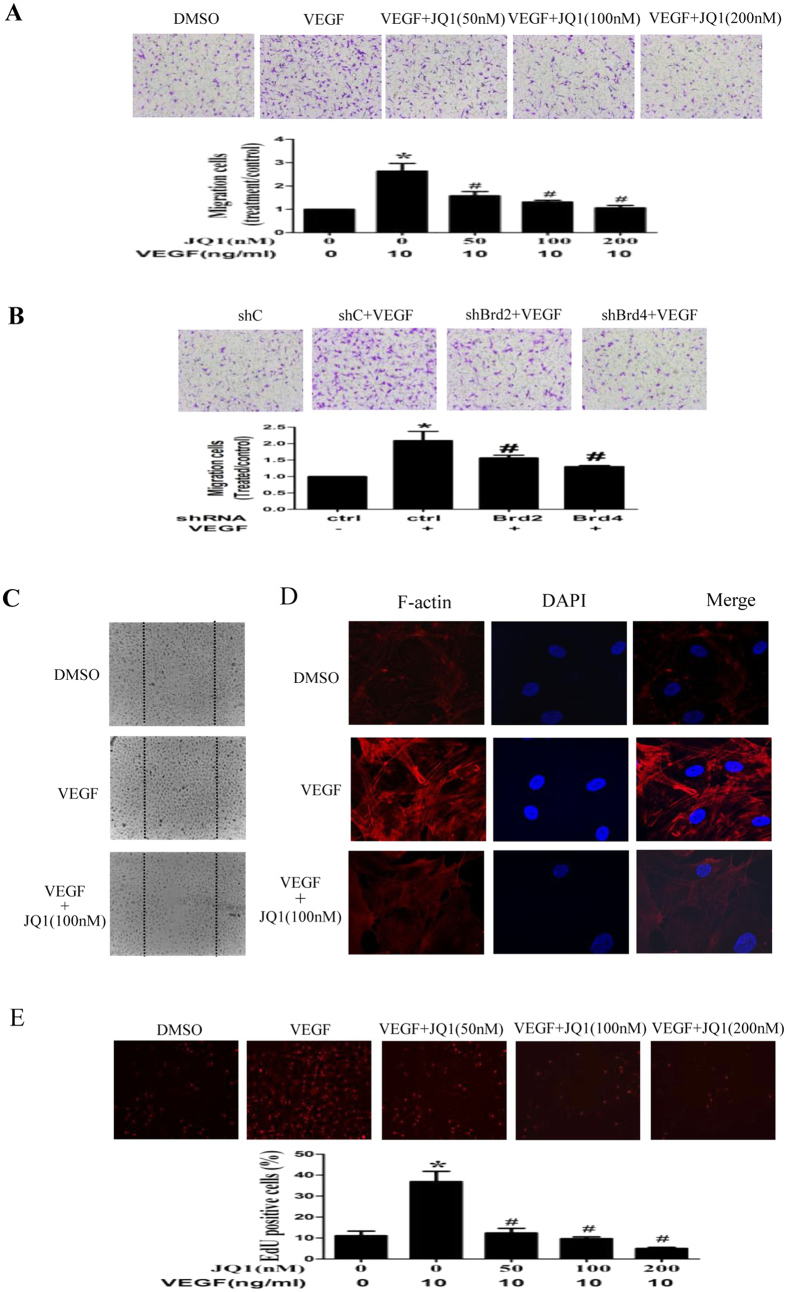
Effect of BET bromodomain inhibition on migration, cytoskeleton alterations, and proliferation of HUVECs. **(A**,**B**) Migration was performed in a Boyden chamber and chemotaxis was quantified by counting migrated cells. Photo images of migration of HUVECs pretreated with various concentrations of JQ1 (**A**) or transfected with Brd2 shRNA or Brd4 shRNA or control shRNA (shC) (**B**) and then stimulated with VEGF (10 ng/mL) or DMSO. (**C**) Effect of JQ1 on wound migration of HUVECs induced by VEGF (original magnification ×100). Cells migrating beyond the reference line were photographed and counted. (**D)** Effect of JQ1 on alterations of actin cytoskeleton in VEGF-induced HUVECs. The cells were pretreated with DMSO or JQ1 (100 nM) for 6 h, and stimulated with VEGF (10 ng/mL) for 12 h. F-actin (red) and nuclei (blue) were stained with phalloidin and DAPI, respectively. Representative images from 3 independent experiments. (**E**) Effect of JQ1 on proliferation of HUVECs. The cells were pretreated with DMSO or various concentration of JQ1 and then stimulated with VEGF (10 ng/mL) for 24 h. EdU incorporation was used to assess proliferation of cells. Representative images from 3 independent experiments. All values represent mean ± SD. *P < 0.05 vs. control, ^#^*P* < 0.05 vs. VEGF.

Migration of endothelial cells plays a key role in angiogenesis. Therefore, we determined the effect of BET inhibition on VEGF-induced migration of HUVECs during chemotaxis. As shown in Fig. 3A, JQ1 treatment markedly inhibited VEGF-induced migration of HUVECs. To confirm the role of BET proteins in regulating migration of ECs, HUVECs were transfected with specific shRNA for Brd2 or Brd4. Western blot analysis revealed that transfection with Brd2 shRNA or Brd4 shRNA markedly inhibited protein expression of Brd2 or Brd4 (Fig. 3B). We also observed that transfection with Brd2 shRNA or Brd4 shRNA suppressed HUVEC migration (Fig. 3C). HUVEC migration was also evaluated in a wound healing assay in the presence or absence of VEGF. Cell movement into the wounded area was detected with light microscopy. As expected, the number of cells that migrated increased in response to VEGF compared with serum-free media. However, JQ1 treatment suppressed migration in response to VEGF (Fig. 3D). Transfection with Brd2 shRNA or Brd4 shRNA was also effective in controlling cell migration (data not shown). These results reveal an important role of BET in regulating angiogenesis through, at least in part, controlling EC migration.

Dynamic reorganization of the actin cytoskeleton is critical for VEGF induction of EC migration^29^. Therefore, we investigated whether BET inhibition regulates the reorganization of the actin cytoskeleton in VEGF-induced HUVECs. As shown in Fig. 3D, treatment with JQ1 affected the number or intensity of F-actin stress fibers in VEGF-treated HUVECs. These results suggest that BET proteins control EC migration by regulating reorganization of the cytoskeleton.

EC proliferation is also an important step for angiogenesis. Therefore, we investigated the effect of BET inhibition on VEGF-induced HUVEC proliferation, which was measured by EdU incorporation. As shown in Fig. 3E, JQ1 suppressed VEGF-induced proliferation of HUVECs. Collectively, these data indicate that the BET bromodomain controls angiogenesis by regulating migration and proliferation of ECs.

### BET inhibition regulates VEGF-induced activation of VEGFR2 and PAK1 in HUVECs

**Figure 4:**
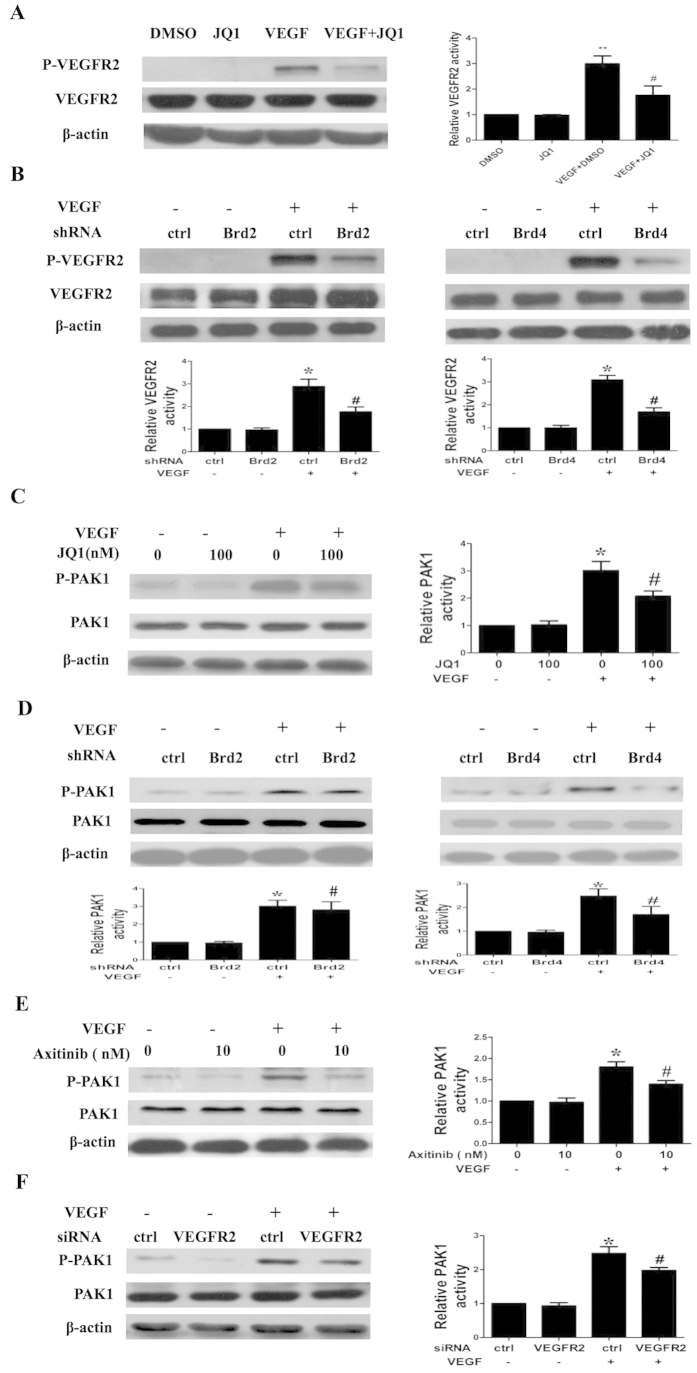
Effect of BET inhibition on VEGF-induced activation of VEGFR2 and PAK1 in HUVECs. (**A**,**B**) Western blot analysis of VEGF-induced phosphorylation of VEGFR2 in HUVECs. The cells were pretreated with DMSO or JQ1 for 6 h or transfected with or without specific Brd2 shRNA (shBrd2) or Brd4 shRNA (shBrd4) or control shRNA (ctrl), and stimulated with 10 ng/mL VEGF for 15 min. (**C**,**D**) Western blot analysis of phosphorylated PAK1 in HUVECs pretreated with DMSO or JQ1 for 3 h (**C**) or transfected with Brd2 shRNA or Brd4 shRNA or control shRNA (**D**) in the presence or absence of VEGF (10 ng/mL). (**E**,**F**) Effect of the VEGFR2 inhibitor axitinib (**E**) or siRNA (**F**) on PAK1 activation. HUVECs were pretreated with axitinib (10 nM) for 1 h or transfected with VEGFR2 siRNA and then stimulated with 10 ng/mL VEGF for 15 min. Densitometry was performed and fold change of protein expression is shown in the right (for JQ1 or axitinib or siRNA) or lower panel (for shRNA). All values represent mean ± SEM. *P < 0.05, **P < 0.01 vs. DMSO or control siRNA or control shRNA, ^#^P < 0.05 vs. VEGF.

VEGFR2-mediated signals play important roles in VEGF-induced angiogenesis. Therefore, we evaluated the effect of BET inhibition on activation of VEGFR2. As shown in Fig. 4A, JQ1 suppressed VEGFR2 phosphorylation, but did not affect VEGFR2 protein expression. We also observed that transfection with Brd2 or Brd4 shRNA reduced VEGFR2 phosphorylation (Fig. 4B). These data suggest a role of BET proteins in controlling VEGF-stimulated EC functions through regulation of VEGFR2 activation.

PAK1, a potential mediator of the Rac1/Cdc42 signaling pathway, is involved in regulating endothelial migration and angiogenesis^13^,^15^. Therefore, we investigated the role of BET proteins in regulating PAK1 activation in response to VEGF. We found that JQ1 treatment inhibited phosphorylation of PAK1 in VEGF-treated HUVECs (Fig. 4C). Transfection with Brd2 or Brd4 shRNA also decreased VEGF-induced PAK1 phosphorylation (Fig. 4D). Furthermore, we found that the VEGFR2 inhibitor axitinib (10 nmol/L) suppressed VEGF-induced phosphorylation of PAK1 (Fig. 4E). To confirm the role of VEGFR2 in regulating PAK1 activation, HUVECs were transfected with specific siRNA for VEGFR2. We found that transfection with VEGFR2 siRNA downregulated PAK1 phosphorylation (Fig. 4F). These results suggest an inhibitory effect of BET suppression on angiogenesis is associated with blockade of VEGFR2/PAK1 activation.

### BET inhibition regulates VEGF-induced activation of eNOS in HUVECs

**Figure 5:**
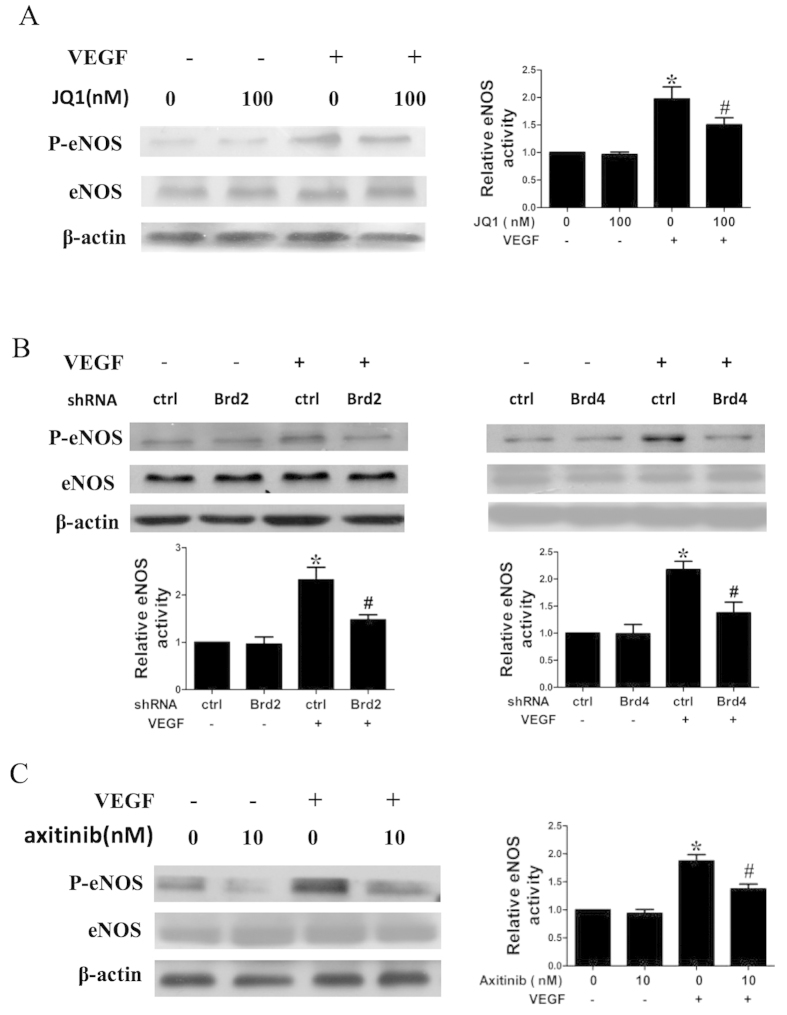
Effect of BET inhibition on VEGF-induced activation of endothelial nitric oxide synthase (eNOS) in HUVECs. **(A**) Western blot analysis of VEGF-induced activation of eNOS in HUVECs. The cells, which were pretreated with DMSO or JQ1 for 6 h, were stimulated with 10 ng/mL VEGF for 15 min. (**B**) HUVECs were transfected with or without specific Brd2 shRNA (shBrd2), Brd4 shRNA (shBrd4), or control shRNA (ctrl), and were then stimulated with VEGF (10 ng/mL) for 15 min. (**C**) Effect of the VEGFR2 inhibitor axitinib on eNOS activation. HUVECs, which were pretreated with or without axitinib (10 nM) for 1 h, were stimulated with 10 ng/mL VEGF for 15 min, and then lysed and analyzed by western blotting. Densitometry was performed and fold change of protein expression is shown in the right (for JQ1 or axitinib) or lower panel (for shRNA). All values represent mean ± SEM. *P < 0.05 vs. DMSO or control shRNA, ^#^*P* < 0.05 vs. VEGF.

Since eNOS also plays a critical role in VEGF-induced angiogenesis and vascular permeability^16^, we evaluated the effect of BET inhibition on eNOS activity (Fig. 5A). We found that JQ1 suppressed phosphorylated eNOS, but not total eNOS. We also observed that transfection with Brd2 or Brd4 shRNA reduced eNOS phosphorylation (Fig. 5B). Furthermore, we demonstrated the inhibitory effect of the VEGFR2 inhibitor axitinib (10 nmol/L) on phosphorylated eNOS (Fig. 5C).

### Inhibition of VEGFR2 and PAK1 activation suppressed VEGF-induced migration, proliferation, and permeability of HUVECs

**Figure 6:**
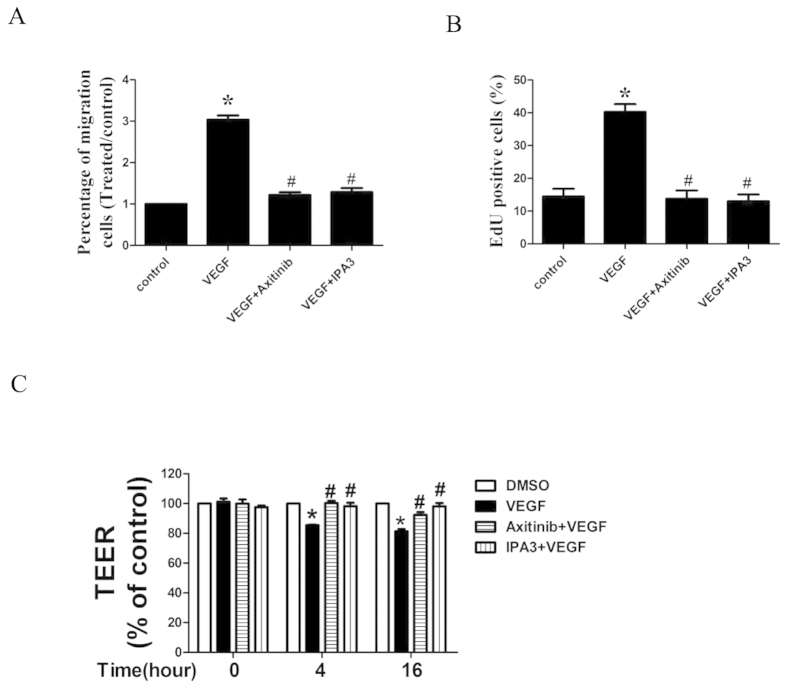
Effect of axitinib and IPA3 on VEGF-induced migration, proliferation, and *in vitro* permeability of HUVECs. **(A**) Effect of VEGFR2 inhibitor axitinib and PAK1 inhibitor IPA3 on HUVEC migration. HUVECs were pretreated with axitinib (10 nmol/L) or IPA3 (30mg/mL) for 3 h and then stimulated with VEGF (10 ng/mL) or 0.1% DMSO (as a control). Migration was performed in a Boyden chamber and chemotaxis was quantified by counting migrated cells. (**B**) Effect of axitinib and IPA3 on HUVEC proliferation. The cells were pretreated with DMSO or axitinib (10 nmol/L) or IPA3 (30 μM) for 1 h and then stimulated with VEGF (10 ng/mL) for 24 h. EdU incorporation was used to assess proliferation of cells. (**C**) Effect of axitinib and IPA3 on permeability of HUVECs *in vitro*. Electrical resistance of a monolayer of HUVECs was measured by transendothelial electrical resistance (TEER). HUVECs were cultured to confluence in 24-well Transwell clear polyester membrane cell culture chambers, treated with DMSO (as a control) or axitinib (10 nmol/L) or IPA3 (30 μM) for 1 h, and then stimulated with VEGF or PBS for 4 h or 16 h. The experiments were carried out in triplicate in 3 separate experiments. All values represent mean ± SEM. *P < 0.05 vs. control, ^**#**^*P* < 0.05 vs. VEGF.

First, we evaluated the effect of the VEGFR2 inhibitor axitinib or the PAK1 inhibitor IPA3 on VEGF-induced migration of HUVECs. As expected, treatment with axitinib or IPA3 decreased VEGF-induced migration (Fig. 6A). Second, we demonstrated that axitinib or IPA3 inhibited proliferation of HUVECs (Fig. 6B). In addition, we found an inhibitory effect of axitinib or IPA3 on VRGF-induced *in vitro* permeability (Fig. 6C).

## Discussion

In the present study, we demonstrated for the first time that BET bromodomain inhibition suppressed VEGF-induced angiogenesis and endothelial permeability *in vitro* and *in vivo*. Furthermore, BET inhibition suppressed proliferation, migration, and reorganization of the actin cytoskeleton in HUVECs. We also showed that BET inhibition suppressed VEGF-induced endothelial VEGFR2/PAK1 activation. Inhibition of activation of VEGFR2 and PAK1 reduced migration and proliferation of HUVECs. We also demonstrated the involvement of BET inhibition in regulating VEGF-induced eNOS activation. In light of these findings, our study identifies the BET bromodomain as a novel regulator of angiogenesis and endothelial permeability.

Angiogenesis is involved in physiological processes such as development and wound healing, as well as in pathological states such as cancer, diabetes, and inflammatory diseases. Anti-angiogenesis is considered a promising strategy for treatment of cancer and several other disorders. Although a recent study indicates an inhibitory effect of Brd4 in controlling endothelial inflammation and atherogenesis by regulating NF-κB activity^26^, the role of BET proteins in controlling angiogenesis is still unclear. In this work, we demonstrated for the first time that BET inhibition suppressed VEGF-induced *in vitro* tube formation and *in vivo* angiogenesis using mouse Matrigel plugs and hyperoxic-induced retinal neovascularization, which suggests an important role of the BET bromodomain in controlling angiogenesis. BET inhibition might be a novel strategy for treatment of angiogenesis-related disorders.

EC barrier integrity is critical to tissue and organ function. Increased endothelial permeability by inflammatory mediators may result in local swelling and edema. Persistent, unresolved endothelial hyperpermeability also underlies the origin and development of angiogenesis. Therefore, conservation of vascular EC barrier integrity has the potential for great clinical impact. In the present study, we determined that JQ1 attenuated VEGF-induced endothelial hyperpermeability *in vitro* and *in vivo*, which suggests an effect of endothelial BET inhibition on decreasing vascular hyperpermeability.

Angiogenesis is a multi-step and complex process that relies on the activation of endothelial cells followed by their migration, proliferation, and morphological differentiation into capillary tubes. Migration and proliferation of endothelial cells in response to angiogenic growth factors such as VEGF play essential roles in this process. In the present study, we showed that BET inhibition with JQ1 or shRNA suppressed VEGF-induced migration and proliferation, which suggests that the BET bromodomain is involved in VEGF-induced angiogenesis by controlling migration and proliferation of ECs. Consistent with our results, other authors have also shown that Brd4 inhibition suppresses migration and proliferation in other cell lines^30^.

VEGF can bind 3 different tyrosine kinase receptors: VEGFR1 (Flt-1), VEGFR2 (Flk-1-KDR), and VEGFR3. VEGFR2 is considered the predominant mediator of VEGF-induced angiogenic signaling. Binding of VEGF-A to VEGFR2 results in VEGFR2 phosphorylation and induces a cascade of different signaling pathways that control angiogenesis and endothelial permeability^31^,^32^,^33^. As a critical mediator of VEGF-induced angiogenesis, in recent years VEGFR2 has been considered a novel important target to inhibit tumor-induced angiogenesis^34^. Therefore, we focused the effect of BET inhibition on VEGFR2-mediated signaling in this work. We found that BET inhibition suppressed VEGF-induced VEGFR2 phosphorylation, but it did not affect VEGFR2 protein expression. The VEGFR2 inhibitor axitinib also decreased VEGF-induced migration and proliferation of HUVECs. These findings suggest that modulation of VEGFR2 activation may be a mechanism by which the BET bromodomain regulates VEGF-induced angiogenesis and permeability. However, the detailed mechanism(s) of action for the BET inhibitory effect on VEGF signaling remains unclear. Previous studies have shown that BET bromodomains localize genome-wide to promoter and enhancer regions^22^,^35^,^36^,^37^. It has been reported that BET inhibitors contribute to suppression of inflammation due to a direct negative action on the transcription of NF-κB target genes in macrophages^22^ and ECs^25^. However, this is not a unique model to explain the mechanism(s) of action for BET inhibition since a recent study indicates that nuclear BET proteins influence cytoplasmic IKK signaling^38^. These data suggest the complexity of mechanism(s) of action for the BET bromodomain in regulating cellular processes. It is well known that binding of VEGF to VEGFR2 induces receptor dimerization, activation of the kinase and autophosphorylation of tyrosine residues, which occurs in cytoplasm of endothelial cells. On the other hand, Brd proteins are highly enriched in nuclear super enhancers (SE), and BET inhibitors such as JQ1 disrupt the formation of the chromatin complexes essential for many genes expression through interfering with binding of Brd proteins to acetylated histones. Thus, this indicates that nuclear located Brd proteins regulate cytoplasmic VEGFR2 activation, most likely, in an indirect manner in VEGF-induced ECs. That is to say, suppression of BET inhibitor in VEGFR2 activation might be through downregulating transcription of genes encoding signaling proteins that function in VEGFR2 phosphorylation. Another possibility is that Brd proteins may mediate “inside-out” signaling from the nucleus to the cytoplasm that regulates VEGF-induced VEGFR2 phosphorylation. However, we did not completely rule out the possibility that Brd proteins might shuttle out of the nucleus and to interact with directly with acetylated VEGFR2 or other acetylated proteins in the cytoplasm to regulate signaling pathways that control VEGFR2 activation in ECs since a recent study shows that VEGFR2 acetylation, under dynamic control of the acetyltransferase p300 and two deacetylases HDAC5 and HDAC6, positively regulates receptor phosphorylation and maintains receptor activity upon prolonged VEGF stimulation^39^. This possibility seems to be little because we did not observe the VEGF-induced translocation of Brd4 from nucleus to cytoplasm, measured by immunofluorescence (data not shown). In the future, it is necessary to explore direct evidence as to how the BET bromodomain modulates VEGFR2 phosphorylation. In addition to VEGFR2, several other factors, including VEGFR1/3, neuropilins (NPs), ephrin-B2 and VE-cadherin, is also involved in modulating VEGF signaling. Thus, we determined the effect of BET inhibitor on the expression of these molecules. However, we found that JQ1 treatment resulted in slight but not significant reduction of the expression of VEGFR1, VEGFR 3, neuropilins (NPs), ephrin-B2 and VE-cadherin (Fig. S2).

PAK1 has been identified as a potential modulator of endothelial cell migration, permeability, and angiogenesis^13^,^15^. A recent report shows that PAK1 is a downstream mediator of VEGFR2 in controlling tumor migration and invasion^40^. This prompted us to investigate whether BET bromodomain inhibition affects PAK1 activation through VEGFR2 signaling. Our results showed that BET inhibition suppressed VEGF-induced PAK1 activation in HUVECs. We also demonstrated that VEGF-induced PAK1 activation was decreased by inhibition of VEGFR2 phosphorylation with a specific inhibitor or siRNA transfection, which suggests that the BET bromodomain controls angiogenesis and endothelial permeability by targeting VEGFR2/PAK1 signaling. In addition, consistent with previous findings^41^,^42^, we also showed that the PAK1 inhibition reduced VEGF-induced migration and proliferation of HUVECs. Therefore, our data suggest that the BET bromodomain controls VEGF-induced angiogenesis through, at least in part, regulating VEGFR2/PAK1-mediated migration and proliferation of ECs.

In addition, previous studies have shown the involvement of eNOS in VEGF-induced angiogenesis and vascular permeability^16^,^43^. In this work, we found that BET inhibition suppressed VEGF-induced phosphorylation of eNOS, but not total eNOS. A VEGFR2 inhibitor also blocked eNOS activation in VEGF-stimulated HUVECs. These data suggest that, in addition to the PAK1 pathway, BET inhibition also modulates VEGFR2-mediated eNOS activity in VEGF-induced ECs.

In summary, we have identified the BET bromodomain as a target that regulates angiogenesis and permeability by regulating VEGFR2-mediated PAK1 and eNOS signaling. Strategies aimed at inhibiting the BET bromodomain may provide a novel therapeutic approach to limiting angiogenesis-related diseases.

## Methods

### Reagents and antibodies

VEGF165 was obtained from R&D Systems (R&D Systems, Minneapolis, MN). Endothelial Basal Medium 2 (EBM2) was purchased from Lonza (Walkersville, MD). Fetal bovine serum (FBS), antibiotics, trypsin-EDTA, phosphate buffered saline (PBS), and other reagents for cell culture were purchased from Invitrogen (Carlsbad, CA, USA). BET inhibitor JQ1, VEGFR2 inhibitor axitinib and PAK1 inhibitor IPA3 were obtained from Selleck Chemicals.β-actin antibody was purchased from Sigma (St. Louis, MO, USA). The VEGFR2, and phospho-VEGFR2 antibodies were purchased from Cell Signaling. The anti-phospho-PAK1, anti-PAK1, anti-phospho-eNOS and anti-eNOS antibodies were obtained from Abcam (Cambridge, MA, USA).

For *in vitro* cellular assays, JQ1, IPA3 and axitinib were diluted in dimethyl sulfoxide (DMSO), and 0.1% DMSO was used as control. For *in vivo* experiments, according to previous report^27^, a stock of 100 mg/ ml JQ1 in DMSO was 20-fold diluted by dropwise addition of a 10% 2-hydroxypropyl-β- cyclodextrin carrier (Sigma) under vortexing, harvesting a 5 mg/ ml final solution. Mice were intraperitoneally or intradermally injected daily with freshly diluted JQ1 (50 mg/ kg/d) or a similar volume of carrier containing 5% DMSO.

### Cell culture

Human umbilical vein endothelial cells (HUVECs) were isolated from fresh umbilical cords after treatment with collagenase. HUVECs were cultured in EBM2 media supplemented with 0.1% hEGF, 0.1% GA-1000 (Gentamicin, Amphotericin-B), 0.1% ascorbic acid, 0.1% R3-IGF-1, 0.1% Heparin, 0.4% hFGF-B, 0.1% VEGF, 0.04% hydrocortisone and 2% FBS, and used at passages 4 to 6.

### Retrovirus generation and transduction

Brd2, Brd4, or the control was silenced in ECs by lentiviral shRNA. The sequences of these shRNA oligonucleotides are listed in supplementary Table 1 (Table S1). To generate virus, Lipofectamine 2000 (Invitrogen) was used to transfect 293T cells with plasmids encoding shRNA targeting Brd2, Brd4, or the control together with CMV-dR8.2 packaging and CMV-VSVG envelope plasmids. After 48 h and 72 h, transfection supernatants containing virus were collected and used to infect ECs in the presence of 8 μg/mL polybrene.

### Transfection of siRNA

For transfection of VEGFR2 siRNA (Santa Cruz), HUVECs were cultured in 12-well plates. A transfection mixture of 100nM siRNA and 10 mg/ml lipofectin in serum-free medium was added to medium-aspirated cells for 4 h. The cells were then incubated with complete DMEM containing 10% FBS for 48 h before experiments.

### Tube formation assay

The anti-angiogenesis ability of JQ1 *in vitro* was evaluated using a tube formation assay. Briefly, a 96-well plate was preincubated with Matrigel at 37 °C for 50 min. HUVECs suspended in EBM2 medium were then seeded onto the Matrigel. Next, they were pretreated with 0.1% of DMSO (as the control) or various concentrations (50, 100, and 200 nM) of JQ1 for 6 h and then stimulated with or without VEGF165 (10 ng/mL). To rule out a nonspecific effect of the small molecule inhibitor, we utilized the shRNA interference technique to selectively reduce Brd2 and Brd4 expression. After 72 h of transfection with Brd2 shRNA (shBrd2) or Brd4 shRNA (shBrd4) or control shRNA (shC), HUVECs were seeded onto the Matrigel. The cells were allowed to form tubes for 6 h at 37 °C, and tube formation was assessed with a ZEISS digital microscope using low-power fields (100×). The assay was replicated 3 times.

### Matrigel plug assay

Matrigel (BD Biosciences, CA, USA) was subcutaneously injected either alone or mixed with VEGF (500 ng/mL) in a total volume of 500 μL into the ventral flanks of 7–8-week-old male null mice. Thereafter, these animals were treated with a daily dose of 50 mg/kg of JQ1 by intraperitoneal injection for 10 d. On day 11, the Matrigel plugs were removed and the Matrigel sections were stained with either H&E or Masson’s trichrome, or immunohistochemistry was performed using CD34 rabbit polyclonal antibody (Abcam, Cambridge, MA, USA).

### Oxygen-induced retinopathy

Newborn C57BL/6J mice were randomly assigned to hyperoxia-normoxia (n = 16) and normoxia (n = 8) groups. At postnatal day (P) 7, hyperoxia-normoxia group mouse pups were exposed to hyperoxia (75 ± 0.5% oxygen) for 5 d (from P7 to P12). After hyperoxic exposure, P12 mice were returned to room air (21% oxygen) and were randomly divided into 2 groups. One group was intraperitoneally administrated with JQ1 (50 mg/kg/d) (n = 8) and the other group was treated with DMSO (as a control, n = 8). At P17, all mice were humanely euthanized and their eyes were enucleated and fixed in 10% formalin in PBS. The eyes were then embedded in paraffin and stained with H&E. The number of blood vessels from each group were calculated under light microscopy and expressed as the mean ± SEM.

### Transendothelial electrical resistance (TEER)

Electrical resistance of the confluent HUVEC monolayer was measured using a Millicell-Electrical Resistance instrument (Millicell-ERS; Millipore, Billerica, MA, USA). HUVECs were cultured to confluence in 24-well Transwell clear polyester membrane cell culture chambers (Corning, Corning, NY), treated with JQ1 or DMSO for 6 h, and then stimulated with VEGF or PBS for 4 h or 16 h. For measurements, both apical and basolateral sides of the endothelial cells were bathed with Hank’s balanced salt solution. Electrical resistance was recorded from probes inserted into the buffer inside and outside until similar values were reproducible on 3 consecutive measurements. The measured potential difference between the upper and the lower wells was used to calculate electrical resistance (Ω/cm^2^). TEER values were then calculated by subtracting the inherent resistance of the filter and the bathing solution.

### Miles permeability assays

Athymic nude mice were pretreated intradermally with DMSO or JQ1 for 5 d. VEGF (100 ng in 50 μL/site) or PBS was injected intradermally in the dorsal skin and the ear. After 15 min, Evans blue (100 mL, 0.5% in 0.9% NaCl, Sigma) was injected through the tail vein and was allowed to circulate for 45 min. Mice were killed and skin and ear samples were dissected, photographed, and placed in formamide (Sigma) at 56 °C for 48 h to extract Evans blue. The amount of Evans blue in each sample was read by spectrophotometry at 620 nm.

### Chemotaxis assay

A Transwell assay was conducted using the Boyden chamber method in 24-well plates with inserts of 6.5 mm diameter and 8μm pore size (Transwell, Corning). The bottom chambers were filled with 600 μL EBM-2 medium containing VEGF. The top chambers were seeded with 200 μL EBM-2 medium (without growth factors and FBS) and HUVECs with JQ1 or HUVECs transfected with shRNA for 24 h. After 8 h, the non-migrating cells were removed from the top chamber by a cotton swab. The migrated cells on the bottom side of the membrane were fixed in methanol and stained with 0.1% crystal violet. Images were taken using a ZEISS digital microscope and the stained cells were counted. The assays were replicated 3 times.

### Wound healing assay

HUVECs were seeded in 24-well plates and grown to nearly 100% confluency. The cells were then scratched with a pipette tip to create wounds. Next, cells were treated with VEGF (10 ng/mL) in the presence or absence of JQ1 (100 nM) in serum-free medium. Randomly chosen fields were photographed at 10× magnification with an inverted microscope, and the images were taken at identical locations at the indicated time points.

### HUVEC proliferation assays

5-Ethynyl-2′-deoxyuridine (EdU) is a thymidine analogue that is incorporated into replicating DNA when cells are dividing and is used to label proliferating cells. HUVECs were trypsinized, counted, and seeded onto 96-well plates at a density of 1 × 10^4 ^cells/well. HUVECs were grown to 80% confluence and were pretreated with or without JQ1 (100 nmol/L) for 6 h prior to incubation without or with VEGF for 24 h. Cell proliferation was then measured using a Cell-Light EdU DNA Cell Proliferation Kit according to the manufacturer’s instructions. Each assay was replicated 3 times.

### RNA isolation and quantitative real-time polymerase chain reaction (qRT-PCR)

Total RNA was extracted using TRIzol (Sigma) and reverse transcribed to cDNA using a miScript Reverse Transcription Kit. qRT-PCR analysis for the expression of Brd2, Brd3, Brd4, and Brdt was performed on cDNA using a QuantiTect SYBR Green RT-PCR Kit on a StepOnePlus TM Real-Time PCR System (Applied Biosystems). Relative mRNA expression was normalized to the expression of GAPDH. The RT-PCR primers used are listed in supplementary Table 2 (STable 2).

### Western blot analysis

Cells were lysed with cell lysis buffer (CST) for 15 min on ice, and lysates were centrifuged for 15 min at 4 °C. Supernatants were then incubated with 2× Laemmli sample buffer (Sigma) at 100 °C for 5 min. Next, equal amounts of samples were then separated with SDS-PAGE gel, transferred to NC membranes, and immunoblotted with the indicated antibodies: anti-VEGFR2, anti-phospho- VEGFR2, anti-phospho-PAK1, anti-PAK1, anti-phospho-eNOS, and anti-eNOS.

### Immunohistochemical analysis

Matrigel plugs were fixed overnight in 10% formalin, paraffin-embedded, cut into 5 μm sections, and placed on 3-aminopropyltriethoxysilane-coated slides. The sections were then deparaffinized with xylene and rehydrated with ethanol, followed by antigen retrieval using microwave heating. Endogenous peroxidase activity was then inhibited by incubating the sections in 1% hydrogen peroxide for 30 min. The sections were then incubated with primary antibodies against CD34 (rabbit polyclonal to CD34, 1:100) (Abcam, Cambridge, UK) at 4 °C overnight. The samples were then washed 3 times with PBS for 5 min each and incubated with the respective horseradish peroxidase (HRP)-conjugated secondary antibodies and substrates, and counterstained with hematoxylin.

### Confocal laser scanning fluorescence microscopy

HUVECs on glass coverslips at 70–80% confluence were treated with JQ1 or DMSO for 6 h and then stimulated with VEGF for 12 h. They were then fixed with paraformaldehyde and permeated with 0.1% TritonX-100 in PBS. For detection of F-actin, cells were incubated with phalloidin overnight. Cells were then incubated with DAPI and the coverslips were mounted on glass slides with antifade mounting media and examined using a confocal fluorescence microscope (Zeiss LSM710).

### Statistical analyses

The data are expressed as mean ± SEM. Student’s *t* test or one-way analysis of variance was used to evaluate differences between experimental groups using SPSS software. P values less than 0.05 were considered statistically significant.

### Study approval

The human study protocol was approved by the Medical Ethical Committee of the First Affiliated Hospital at Sun Yat-sen University and was conducted according to the *recommendations of the Declaration of Helsinki*. All patients provided informed consent to participate in the study. The experimental protocols for animal care and use were reviewed and approved by the Animal Care and Ethics Committee of the First Affiliated Hospital at Sun Yat-sen University according to the *Guide for the Care and Use of Laboratory Animals*, which was published by the US National Institutes of Health. All protocols were conducted in accordance with ethical guidelines and approved by the Animal Welfare Committee of First Affiliated Hospital at Sun Yat-sen University.

## Additional Information

**How to cite this article**: Huang, M. *et al*. BET Bromodomain Suppression Inhibits VEGF-induced Angiogenesis and Vascular Permeability by Blocking VEGFR2-mediated Activation of PAK1 and eNOS. *Sci. Rep.* **6**, 23770; doi: 10.1038/srep23770 (2016).

## Supplementary Material

Supplementary Information
