# Supplementary material for: BET Bromodomain Suppression Inhibits VEGF-induced Angiogenesis and Vascular Permeability by Blocking VEGFR2-mediated Activation of PAK1 and eNOS
Source: Sci Rep. 2016 Apr 5;6:23770. doi: 10.1038/srep23770 (PMC4820704; doi:10.1038/srep23770)
Supplement: Supplementary Information [file srep23770-s1.pdf]

SUPPLEMENTARY INFORMATION

BET Bromodomain Suppression Inhibits VEGF-induced Angiogenesis and Vascular Permeability by Blocking VEGFR2-mediated Activation of PAK1 and eNOS

Mingcheng Huang1, #, Qian Qiu 1, #, Youjun Xiao 1, #, Shan Zeng1, Mingying Zhan2, Maohua Shi1, Yaoyao Zou 1, Yujin Ye 1, Liuqin Liang 1 , Xiuyan Yang 1, Hanshi Xu1.

Supplementary Figure S1

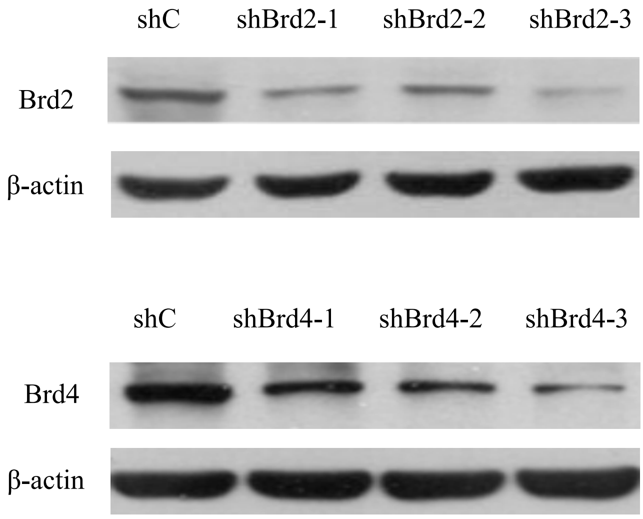

Supplementary Figure S1. Targeted depletion of Brd2 and Brd4 in HUVECs. HUVECs were transfected with Brd2 shRNA (shBrd2) or Brd4 shRNA (shBrd4) oligonucleotides or control shRNA (shC). After 72 h of transfection, cells were lysed, and expression of Brd2 or Brd4 was assessed by western blot analysis.

Supplementary Figure S2

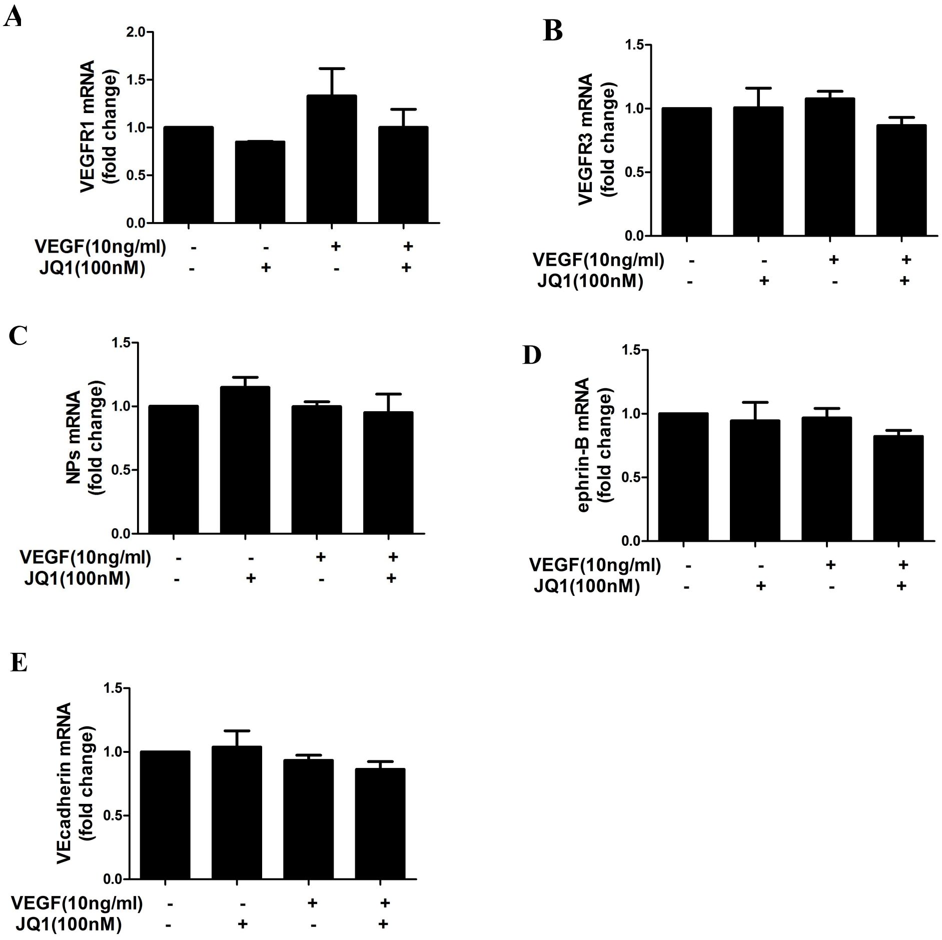

Supplementary Figure S2. Effect of BET inhibition on VEGF-induced expression of VEGFR1, VEGFR 3, neuropilins, ephrin-B2 and VE-cadherin in HUVECs. The cells were treated with DMSO or JQ1 (100 nM) for 6 h, and then stimulated with VEGF (10 ng/mL) for 12 h. The expression of VEGFR1 (A), VEGFR3 (B), neuropilins (C), ephrin-B2 (D) and VE-cadherin (E) was measured by qRT-PCR analysis. The data represent mean ± SEM from 3 independent experiments.

Supplemental Table

STable 1 The sequences of Brd shRNA oligonucleotides

		Oligo Sequence
**Brd2** shRNA	Forward	AATTCCCTGCCTACAGGTTATGATTCTCGAGAATCATAACCTGTAGGCAGGGTTTTTAT
	Reverse	AAAAACCCTGCCTACAGGTTATGATTCTCGAGAATCATAACCTGTAGGCAGGG
**Brd4** shRNA	Forward	AATTCCTGGAGATGACATAGTCTTACTCGAGTAAGACTATGTCATCTCCAGGTTTTTAT
	Reverse	AAAAACCTGGAGATGACATAGTCTTACTCGAGTAAGACTATGTCATCTCCAGG
**Scramble** shRNA	Forward	AATTCCTAAGGTTAAGTCGCCCTCGCTCGAGCGAGGGCGACTTAACCTTAGGTTTTTAT
	Reverse	AAAAACCTAAGGTTAAGTCGCCCTCGCTCGAGCGAGGGCGACTTAACCTTAGG

STable 2 The sequences of RT-PCR primers

	sequences	
Brd2	Forward	ACTCACCTCTTCAGAACGA ATTG
	Reverse	CATCT TTGGAAGGTTCAGGTTG
Brd3	Forward	ACTGAGAGTGATTGAGAGTGGAC
	Reverse	AACCCTCT GCACCCAGTTTTC
Brd4	Forward	CTCTGGAGTAATGTCACACCTCT
	Reverse	TGTTGGTC CACCTTTCATCTTC
Brdt	Forward	TGTAAAGAAACCTTCCTGCAA
	Reverse	TTTAAAACACAGTATGCCCAA
VEGFR-1	Forward	GAAAACGCATAATCTGGGACAGT
	Reverse	GCGTGGTGTGCTTATTTGGA
VEGFR-3	Forward	CTGGACCGAGTTTGTGGAGG
	Reverse	GTCACATAGAAGTAGATGAGCCG
NPs	Forward	AATCTCATCCTAGTTCTGTCGCT
	Reverse	CTCCTGTCCAATCTGGTTGGG
VEcadherin	Forward	AAGCGTGAGTCGCAAGAATG
	Reverse	TCTCCAGGTTTTCGCCAGTG
ephrin-B	Forward	TATGCAGAACTGCGATTTCCAA
	Reverse	TGGGTATAGTACCAGTCCTTGTC
